# Prefrontal cortex activity during virtual obstacle avoidance and distracted walking: A methodological proof of concept using augmented reality and functional near-infrared spectroscopy

**DOI:** 10.1371/journal.pone.0333622

**Published:** 2025-10-10

**Authors:** L. Maureen Krelove, Anthony Machula, Lauren E. Sergio, George Mochizuki

**Affiliations:** 1 School of Kinesiology & Health Science, Faculty of Health, York University, Toronto, Ontario, Canada; 2 Centre for Integrative and Applied Neuroscience, York University, Toronto, Ontario, Canada; 3 Centre for Vision Research, York University, Toronto, Ontario, Canada; Tokyo Metropolitan Institute of Geriatrics and Gerontology, JAPAN

## Abstract

**Introduction:**

There is an established interplay between gait and attention allocation. Attention during walking is important to reduce instability, process environmental stimuli, and perform simultaneous tasks. It is of critical importance to consider how attention modulation during dual-tasking influences prefrontal cortex (PFC) activity and gait characteristics. However, paradigms probing this relationship are often limited in realism and must balance mobility challenges with practicality. This protocol introduces a novel methodology combining functional near-infrared spectroscopy (fNIRS) and augmented reality (AR) in a complex gait dual-task to validate the use of virtual obstacles to probe for a cortical indicator of altered attention during distracted walking.

**Materials and methods:**

This methodological development study investigated 11 healthy adults (mean age 50.9 ± 5.8 years, 5 female) in an obstacle navigation cognitive-motor dual-task combining fNIRS and AR during navigation of realistic AR-projected 3D virtual obstacles and physical obstacles. The distraction task involved a 5-word recall from a mimicked phone call. Participants performed six experimental tasks: walking; walking + distraction; walking + obstacles (both physical and virtual); and walking + obstacles (both physical and virtual) + distraction.

**Results:**

Intraclass correlations ranged from 0.563 to 0.886 for oxyhemoglobin (O_2_Hb) ratios and gait velocity between virtual and physical obstacles, demonstrating moderate-to-good consistency between methods. Proportional bias in the Bland-Altman plots was observed for O_2_Hb. Participants also demonstrated task-dependent modulation of gait and PFC activity in response to dual task conditions in both tasks.

**Conclusions:**

This combination of technologies elicited task-dependent modulation in PFC activity and gait behaviours in healthy adults, confirming the efficacy of AR-projected obstacles in a cognitive-motor dual-task paradigm. Based on these outcomes, it is likely that this experimental approach will be useful in probing cortical activity changes associated with dual-tasking to inform the relationship between mobility and cognition and characterize behavioural and neural markers of functional mobility.

## Introduction

The allocation of attention to environmental stimuli is an essential component of safe and effective ambulation. Walking in the real world requires attention to be paid to both the motor task and the environment to monitor for any relevant changes to current task contexts or impending balance challenges, and are often done at the same time as a secondary cognitive distractor [1]. In modern society, we face not only the attentional challenges of moving from one location to another, but are often also required to contend with additional tasks including avoiding obstacles, carrying a conversation, or interacting with a mobile device. During these dual tasks, active attention becomes further divided, and may come with a dual-task cost wherein performance on one or both tasks is compromised [2–4]. This established interplay between cognition and locomotion highlights that gait requires attention, and it is known that alterations in cognition can influence the allocation of attentional resources that can compromise stability [1,2]. Understanding the interrelation of gait and cognition from a mobility perspective is essential to mitigate increased fall risk that can occur during distraction. However, paradigms probing this relationship are often limited in real world applicability, ecological validity, and must balance mobility challenges with practicality.

The division of attention required to complete these simultaneous dual tasks relies on the prefrontal cortex (PFC), which has a central role in higher order cognition, including the allocation of attentional resources, and may alter activity depending on task demands and participant population [5]. When attention networks are altered, PFC activity often increases in compensation to try to maintain task performance [6]. Indeed, healthy older adults tend to increase PFC activity during walking dual-tasks and show reduced cognitive performance and gait speed [2]. It is thus of critical importance to consider how attention modulation during dual-tasking influences both PFC activity and the characteristics of movement to inform the relationship between mobility and cognition, identify markers of movement limitations, and characterize neural markers of increased fall risk.

Alterations in PFC activity during dual-task gait in young and older adults have been probed using functional near-infrared spectroscopy (fNIRS), a form of functional neuroimaging measuring the concentration of cortical oxygenated (O_2_Hb) and deoxygenated (HHb) hemoglobin [7–15]. A challenge during these dual-task gait experiments is balancing the difficulty of the dual-task challenges while maintaining safety and feasibility in a laboratory setting, where one can collect comprehensive performance metrics. Further, relatability of laboratory tasks to daily life is necessary to elicit outcomes as similar as possible to the real-world. Indeed, high task realism contributes to enhanced cognitive and motor performance in older adults during virtual environment tasks [16]. The cognitive task should be difficult enough to challenge healthy attentional systems but not risk harm or stress to the participant. The addition of physical obstacles to the walking path may alter the allocation of attention if obstacles are viewed as physical hazards for trips or falls, and in general, may pose a safety concern in the laboratory environment.

The proposed methodology introduces realistic virtual obstacles, which participants must avoid, in the real environment using augmented reality (AR). O_2_Hb and gait characteristics are examined during distracted obstacle avoidance with virtual obstacles and compared and validated against navigation around comparable physical obstacles. To our knowledge, this is the first time that fNIRS and AR have been combined in an obstacle avoidance cognitive-motor dual-task paradigm. If task-dependent differences are elicited and quantified in this small pilot cohort and virtual obstacles reliability elicit similar levels of PFC activity and gait behaviours as physical obstacles, this safe, non-invasive approach is likely to remain robust in empirical studies examining populations with age- or injury-related attentional impairments.

## Materials and methods

### Equipment

**Fig 1:**
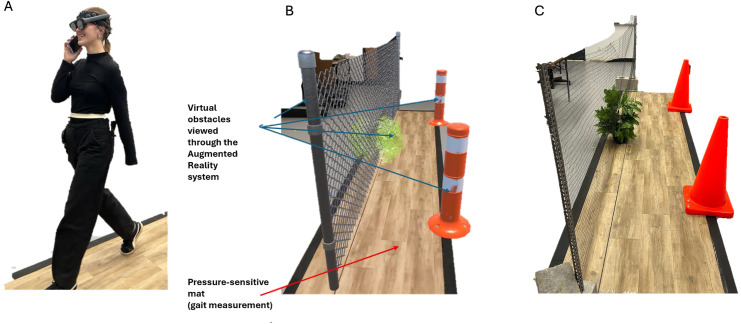
Equipment and paradigm set up. Participant wearing all equipment for the study while walking – the fNIRS is worn on the forehead and the AR is worn attached to a shoulder strap to minimize interference **(A)**. Virtual obstacles (blue arrows) as they appear in the lab environment on the gait mat (red arrow) as projected by AR **(B)**. The position of the physical obstacles matches the position of the virtual obstacles **(C)**.

A portable, non-invasive 8-channel fNIRS unit (Octamon, Artinis Medical Systems, Elst, The Netherlands) was used to measure relative change in cortical blood O_2_Hb and HHb in the dlPFC using light wavelengths of 760nm and 850nm at a 50 Hz sampling rate. Emitting optodes and receivers were 30 mm apart with a tissue penetration depth of 1.5 cm. The modified Beer-Lambert Law was applied to convert optical density into concentrations in real-time using a differential pathlength factor of 6 [17,18]. Concentration changes in micromolar (uM) were collected using OxySoft v.3.2.72. Gait characteristics were measured using a 14 x 3 ft gait mat (ZENO^TM^ Walkway Gait Analysis System, ProtoKinetics, Havertown, PA, USA). Gait data were collected at 120 Hz and analyzed using ProtoKinetics Movement Analysis Software version 5.08C1i6. Virtual objects were projected onto the AR lenses and appeared to participants to be on the gait mat (Fig 1). The lightweight (260 grams) AR headset (MagicLeap 2, MagicLeap, Plantation, FL, USA) and fNIRS unit were able to be worn comfortably on a participant’s head (Fig 1). Custom code was written to integrate three-dimensional objects into the AR space (Unity Technologies, San Francisco, CA, USA). The AR headset also recorded head position and rotation information (1 KHz) in three directions using custom C# code run through Unity in JSON format. Head position data were collected but is not included in the present analyses.

### Methodology

#### Participants.

Healthy adults were recruited from the York University community in Toronto, Canada. To be included in this methodological proof of concept study, participants had to be > 35 years of age with no stroke history, neurological diagnoses, concussion diagnoses and/or symptoms in the past year, and no musculoskeletal injuries or surgeries in the past year that could impact balance or gait. Written informed consent was obtained from all participants prior to beginning the study and this study was approved by the Research Ethics Board at York University (certificate #e2023-198).

#### Experimental design.

**Fig 2:**
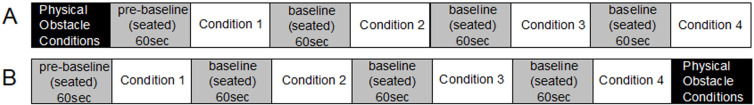
Schematic showing the temporal organization of the pseudorandom study design. Participants either completed the study as per panel A or B**.**

Data collection for fNIRS was separated into active task conditions and rest conditions. All active conditions involved walking at a self-selected, comfortable pace along the gait mat and varied based on obstacle avoidance and distraction. Participants either viewed the room as-is through the AR headset with no obstacles, with the addition of virtual three-dimensional, daily life obstacles on the gait mat, or with physical versions of the AR obstacles in the same location as the virtual obstacles on the gait mat (Fig 1). For active tasks with virtual or physical obstacles, participants were instructed to avoid the obstacles. The distractor task consisted of a “phone call” in which participants played an audio recording of the experimenter providing a five-word list from their smartphone device while holding it up to their ear like a phone call (Fig 1). Words were randomly selected from the Toronto Word Pool for near immediate recall – starting at ten seconds after the end of the recorded word list [19,20]. This created distractor and non-distractor conditions. To ensure consistency of neural activity, the participant held the phone to their ear as though they were on a call for the duration of the active task, even after the recording had ended and they had repeated the word list. A different word list was used for each distractor condition. Taken together, this methodology consisted of six active conditions that throughout the experiment: walking (W); walking + virtual obstacles (WO); walking + distractor (WD); walking + virtual obstacles + distractor (WOD); walking + physical obstacles (WOp); and walking + physical obstacles + distractor (WODp). Virtual and physical obstacle tasks were paired together with order randomized across participants (Fig 2).

Participants first received a full instruction of the data collection process and introduction to the equipment and procedures. Once all equipment was fitted and comfortable, participants sat in a chair for one minute while counting by one from one, this comprised the pre-baseline condition. Simple counting reduced mind wandering to produce a more consistent baseline between participants without the task becoming so difficult that PFC activity would not decrease [8]. This one-minute rest task was repeated between active conditions for a total of four seated baselines. These rest conditions allowed for a physical break and for the PFC hemodynamic response to return to baseline between active conditions. The length of active conditions varied between participants based on walking speed, and the number of times participants needed to walk along the gait mat to obtain enough footfalls (15 footfalls) to calculate gait metrics. In general, each active condition took about one minute with approximately 20 footfalls captured per trial. During active conditions, participants started their walking at a distance 2m behind the mat and continued to a point 2m beyond the mat before turning to continue their walking trial. The added distance allowed for steady state gait to be quantified while acceleration and deceleration took place off the mat. This process was repeated until sufficient footfalls were obtained. Overall, the data collection duration per participant was approximately 15 minutes.

### Data analysis

#### Gait analysis.

Gait data were visually inspected for incomplete footsteps, and any found were removed (usually 3–5 steps) manually so that only complete footsteps in which the entire footfall was on the gait mat were used to compute gait metrics. Gait analysis was performed using the ProtoKinetic Movement Analysis Software to output common spatiotemporal gait metrics. For the purposes of this study, gait velocity was selected as the primary metric of interest. Across dual-task gait studies, the effect of a cognitive task is predominantly shown through gait speed changes [21]. Further, gait velocity is an established measure for sensitively assessing and monitoring functional state and overall health across populations and ages and is even considered to be a “sixth vital sign” [22,23]. Velocity can be considered a functional balance indicator and it tends to decrease with task challenge and attentional requirements [24]. During dual-tasking, gait velocity has shown to be predictive of future falls, suggesting a unique capacity of dual-task walking to highlight and predict decline in gait and mobility behaviours [25–27].

#### fNIRS analysis.

fNIRS analysis was performed in alignment with the guidelines proposed by Pinti et al (2018). First, raw data were visualized in OxySoft to assess signal quality, denoted by the presence of the heart beat at about 1 Hz that confirms a good optode-scalp coupling [28]. Large motion artefacts were minimal, but if present were removed at this stage. Next, raw data were converted to MATLAB using open access code from Artinis Medical Systems (oxysoft2matlab) to convert*.oxy4* files to MATLAB script. fNIRS processing software Homer3 was used to filter the signals.

The present study applied a filtering pipeline modified from Holtzer et al (2011) [11]. First, optical density data were low-pass filtered at 0.1 Hz to remove most high-frequency physiological noise. Then, a PCA was applied to address non-hemodynamic response signals that overlap in the response band. A PCA was selected as a short source-detector separation was not possible with the hardware. PCA is a data-driven approach to separating neurovascular coupling from systemic physiological noise [29]. Once filtered, data were converted to concentration (uM) using the modified Beer-Lambert Law [30].

Filtered data were averaged across all 8 channels and separated based on whether it was an active or rest condition, and, if active, which task it was. Using Spike2 v10 (Cambridge Electronic Design, Cambridge, UK), the walking condition was compared to the three active, dual-task conditions using the modulus. The modulus assesses waveform data by assessing the area under the curve as a positive value for all data. Final data are expressed as a ratio of area-under-the-curve for active:walk concentration of O_2_Hb per condition. Only O_2_Hb ratios were investigated and reported as the O_2_Hb signal is known to: have the strongest coupling with the fMRI-BOLD signal and thus have a high contrast-to-noise ratio than HHb [31]; be a more robust indicator of changes in regional cerebral blood flow than HHb [32–34]; change its levels in accordance with locomotion activities in a way that is not seen with HHb levels [35].

### Statistical analysis

All statistical analyses were conducted using SPSSv29 (IBM, New York, USA) and OriginPro2025 (OriginLab, Northampton, USA). Descriptive statistics were used to characterize participant demographics. To assess task-based modulation of gait behaviour, velocity was examined via a repeated measures ANOVA with condition as the within-subjects factor. Statistical significance was set to p < 0.05.

To test the hypothesis that virtual obstacles elicited the similar levels of PFC activity and gait velocity changes as physical obstacles, reliability analyses were conducted. Agreement between physical and virtual obstacles for PFC activity and gait velocity was assessed using Bland-Altman analysis in OriginLab 2025 [36]. For each outcome, the difference between the two obstacle types per condition (WO/WOp and WOD/WODp) was plotted against the mean of the two conditions and the mean bias and 95% limits of agreement (LoA) were calculated (mean bias ± 1.96 x SD of the differences). Plots were visually inspected for systemic bias and heteroscedasticity. To probe consistency of outcomes between physical and virtual obstacle outcomes, the intraclass correlation coefficients (ICCs) were found for each obstacle condition. Based on Koo & Li, ICC estimates and their 95% confidence intervals were calculated in SPSSv29 based on a mean-rating (k = 2), consistency, two-way mixed-effects model [37]. Magnitude was interpreted as: < 0.5 poor, 0.50–0.75 moderate, 0.75–0.90 good, > 0.90 excellent.

## Results

### Participant demographics

Data from 11 healthy adults were collected to assess feasibility of this proof-of-concept methodology, compatibility of technologies to probe the measures of interest, and confirm the validity and reliability of virtual obstacles in dual-task paradigms. Table 1 shows the demographic information for participants included in this study, as well as their performance on cognitive tasks (WD, WOD, WODp).

**Table 1:** Participant characteristics and dual-task performance.

Group Size	11 (5 female)
**Age (years)**	50.9 ± 5.8
**Height (cm)**	167.2 ± 8.5
**Weight (kg)**	76.2 ± 16.1
**Number of Total Responses**	
WD	4 (3,5)
WOD	4 (3,5)
WODp	4 (3,5)
**Number of Correct Responses**	
WD	4 (3,5)
WOD	4 (2,5)
WODp	4 (1,5)
**Error Rate**	
WD	0% (0%,25%)
WOD	0% (0%,33%)
WODp	0% (0%, 66%)
**Highest level of education**	
Post-Graduate	6
Undergraduate/College	5
High School	0
Fall History	0%
Activities-specific Balance Confidence (ABC)	98 (86,100)

Participant demographics all shown as mean ± standard deviation with the exception of fall history (proportion of participants with recent fall), dual-task responses and ABC score expressed as median (range), and dual-task error rate (% incorrect of total responses).

### PFC activity

**Fig 3:**
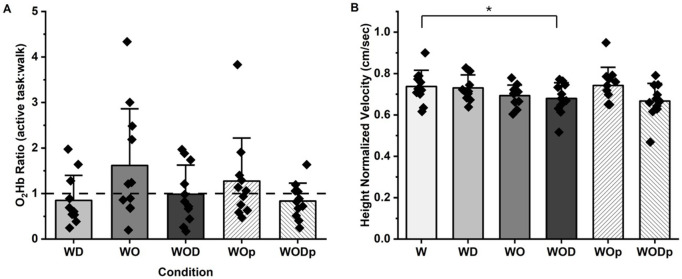
A) PFC activity expressed as a ratio of O_2_Hb concentration across conditions (active task:walk). The black horizontal line at 1 represents the walk value (baseline). B)Height normalized velocity across conditions. * denotes significant difference between W and WOD (p < 0.05).

In general, PFC activity based on blood oxygen concentration (active:walk) shows task dependent modulation as condition requirements changed (Fig 3A). This modulation varied by participant, and in some instances, blood oxygenation levels for an active condition were lower than at baseline. In general, the addition of physical or virtual obstacles (Fig 3; WO, WOD, WOp, WODp) resulted in active:walk ratios greater than the pre-baseline value of 1.

### Gait behaviour

Gait behaviour modulation across conditions was observed (Fig 3B). In general, gait velocity decreased with the addition of a secondary task (F_3,30_ = 7.237, p < 0.001, η^2 ^= 0.420). Post-hoc comparisons showed that the reduction in velocity between W and WOD was statistically significant (p = 0.011) and the reduction between W and WO approached significance (p = 0.057). The additional reduction in velocity from W to WO and then from WO to WOD shows the ability of both virtual and physical obstacles to induce behavioural changes during walking (W vs WO/WOp and WOD/WODp), and that the effect of the dual-task is exacerbated by additional obstacle avoidance (WD vs WOD/WODp).

### Validation of virtual obstacles

**Fig 4:**
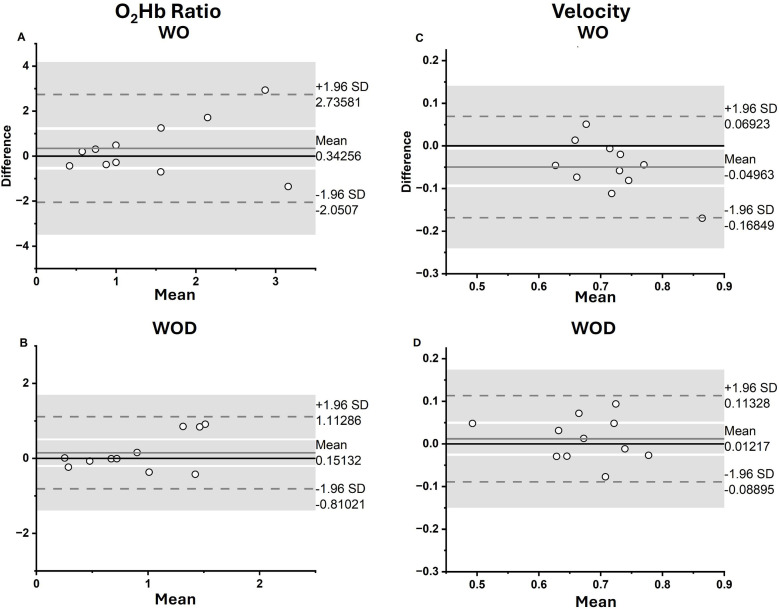
Bland-Altman plots for O_2_Hb Ratio (A, B) and Velocity (C, D).

Virtual, AR-projected obstacles elicited similar neurophysiological and gait velocity changes as physical obstacles during avoidance (Fig 4; Table 2). Bland-Altman and ICC results show that in general, virtual and physical obstacles elicit similar gait and cortical outcomes with good internal consistency between obstacle types. The Bland-Altman plot for O_2_Hb demonstrated rightward funneling/proportional bias (Fig 4A and 4B). This pattern likely contributed to the relatively high BA ratios (Table 2). This was not observed in the Bland-Altman plots for Velocity, which had relatively low ratios.

**Table 2:** Reliability statistics outcomes for virtual and real obstacles.

	Bland-Altman Ratio	ICC [95% CI]
**WO-WOp**		
O_2_Hb Ratio	1.660	0.563 [−0.625, 0.822]
Velocity	0.166	0.766 [0.186, 0.941] *
**WOD-WODp**		
O_2_Hb Ratio	1.055	0.727 [−0.013, 0.972] *
Velocity	0.150	0.886 [0.578, 0.969] *

Bland-Altman analysis outcomes shown as the Bland-Altman ratio for virtual and real obstacles during obstacle navigation with and without cognitive distraction. ICC output for virtual and real obstacles shown as the ICC(3,k) value. * = statistically significant (ICC p < 0.05).

## Discussion

This report presents a novel methodology combining AR and fNIRS during complex dual-task gait to assess the efficacy and reliability of virtual AR projected obstacles in a proof-of-concept cohort of healthy adults. The combination of technologies and experimental paradigm show reliable outcomes and elicit task-based modulation in gait velocity and PFC activity as expected in a gait dual-task. Based on these preliminary outcomes, this approach is likely to remain highly sensitive in populations experiencing age-related changes to attention networks, PFC activity, gait behaviour, and cognitive function.

### PFC activity and cognitive load

The present work represents the first time that AR and fNIRS have been combined to measure PFC activity as a measure of attention during complex gait dual-task. The signals obtained in this paradigm are likely from the dorsolateral PFC (dlPFC), known to be heavily involved with attention allocation during postural tasks. Increased dlPFC oxygenation is indicative of heightened attentional demands [3,7,8,38,39]. Previous work has characterized dlPFC activity using fNIRS during gait dual-task [7,8,11,14,15], gait behaviour during single- and dual-task in young and old adults [40,41], investigated walking metrics as fall predictors in older adults [1,42,43], and assessed the capacity of virtual realities for older adult usage [16]. In general, these studies have identified inconsistent findings as to whether PFC activity increases or decreases during dual tasks [11,13,44–46]. It is notable that in the present study, some participants demonstrated O_2_Hb ratios lower than walking during active task conditions. Despite this, the pattern of task-dependent modulation is consistent such that PFC activity is above baseline during at least one obstacle-avoidance condition. On the one hand, this observation is in line with the literature reporting variability in PFC activity with distraction [11,13,45,46]. On the other, this observation also identifies capacity of the methodology to evoke modulation in PFC activity, even in a group of individuals with variable levels of cortical activity.

Further, dual-task performance in terms of the mean number of responses means did not change with an additional task; however, the number of correct responses decreased with the addition of obstacle avoidance. This decrease was similar for both virtual and physical obstacles, further supporting the ability of virtual obstacles to elicit dual task effects. The task-dependent differences on the word recall correctness also verifies that the presentation of virtual obstacles impacted performance on both the primary and secondary tasks and that these behavioural differences occurred in parallel with differences in brain activity.

### Realism and reliability of virtual obstacles

To the authors’ knowledge, this is the first time that AR has been used to introduce virtual obstacles during gait dual-tasks. One study did employ non-physical laser projected obstacles to resemble potholes to investigate slowing of gait and PFC activity level in young and older adults [47]. However, in this study, obstacles were two-dimensional and limited in realism. The design of both AR and VR environments can incorporate the motor and functional ability of the user to address specific aspects of a perception-action system [48]. AR offers the same customizability and user interactions as virtual reality without creating entirely immersive virtual environments, but rather augmenting physical reality to address needs of both user and experimenter. AR offers the ability to customize physical spaces to facilitate cognitive, sensory, and motor tasks not otherwise possible, such as bringing everyday objects into the laboratory space in a safe, risk-free way.

Crucially, the virtual obstacles elicited similar levels of PFC activity and gait velocity changes as the interaction with physical obstacles. In cases where PFC activity differed between physical and virtual obstacles, virtual obstacles elicited a higher level of PFC activity than physical obstacles. This may indicate the increased salience of the virtual obstacles, or an element of novelty in interacting with augmented reality. In either case, the augmentation of PFC activity during avoidance of virtual obstacles was at least similar, if not higher than activity during avoidance of physical obstacles. One important consideration is that the Bland-Altman plots for O_2_Hb ratio in both conditions demonstrated positive funnelling, indicating heteroscedastic disagreement that increases with higher levels mean activations between conditions. The fact that the ICC for WO-WOp was not statistically significant (p < 0.102) also indicates low cross-obstacle type reliability. Thus, there is some inability to interchange PFC activity levels between obstacle types, especially at much higher levels of PFC activity. This somewhat tempers the interpretation that the virtual and physical obstacles elicit similar levels of PFC activity. However, it is notable that it was interaction with the virtual obstacles that elicited higher activity in those instances in which the difference in O_2_Hb ratio between obstacle types was high. This may indicate that some participants found the AR interaction to be particularly novel with higher salience, rather than ignoring the virtual obstacles altogether.

In terms of gait velocity, there was a downward trend in speed with the addition of any task, and the change in speed for the addition of virtual vs physical obstacles was similar. This reliability in modulating behaviour using virtual obstacles reinforces the feasibility of the paradigm in a mobility context. The reliability of velocity changes in essential for the method to be of use in potentially indicating cognitive or functional state [21,22]. Importantly, there was no interference of the AR system on the fNIRS recording, preprocessing, or analysis steps. The hardware did not cause any participant discomfort, and the combination did not interfere with the requirements of either device during the study. Thus, this novel combination of technologies is likely to remain comfortable, accessible, and simple to apply when the paradigm is applied to other populations and in alternate settings.

Taken together, the virtual obstacles seem to engage behavioural and neurophysiological process in a comparable manner to physical obstacle avoidance. The caveat is that there is some dissociation between measures when the difference in O_2_Hb is high. In these instances, participants tended to demonstrate greater O_2_Hb levels when navigating virtual obstacles. This reinforces the possibility of using AR environments in gait dual-task paradigms to maintain participant safety, increase task realism, and elicit outcomes of interest in the laboratory setting in populations who may experience impairments in balance, mobility or attention.

### Limitations

It should be noted that the dlPFC was the only accessible region for the fNIRS unit used in this study due to limited channel number. It is possible that the signals obtained were not solely from the dlPFC, however this was unlikely based on optode placement and penetration depth. Conversely, the dlPFC may not be the ideal region of interest on its own to measure PFC activity during complex gait dual-task, and inclusion of signals from broader frontal regions may increase reliability of conclusions. However, the dlPFC is known to be heavily involved with attention allocation during postural tasks, and increased dlPFC oxygenation suggests increased attentional demands and reduced cognitive performance in older adults [3,7,8,38,39]. Further, the narrow width of the gait mat and obstacle placement resulted in some important steps in the middle of the walking path to be missed. However, these individual steps would not have had a substantive effect on mean gait velocity calculated across all the steps for a given trial.

## Conclusion

This study introduces the use of virtual AR obstacles alongside fNIRS to reliably investigate mobility, cortical activity, and dual-tasking in a safe and feasible way. Crucially, the paradigm successfully challenges attention, cognition, and gait in healthy adults wherein virtual obstacles elicit the same level of behavioural and neurophysiological changes as physical obstacles. The proposed methodology allows for realism of obstacles unobtainable without AR while maintaining participant safety and practical laboratory feasibility. This pilot study may enable more in-depth investigations to probe for a cortical marker of altered attention during aging. Future work should focus on confirming the sensitivity of the paradigm to detecting age-effects in healthy older adult populations before investigating populations with impairment or injury that could further increase fall risk.
